# Nicotinamide adenine dinucleotides and their precursor NMN have no direct effect on microtubule dynamics in purified brain tubulin

**DOI:** 10.1371/journal.pone.0220794

**Published:** 2019-08-08

**Authors:** Anna Luchniak, Mohammed Mahamdeh, Jonathon Howard

**Affiliations:** Department of Molecular Biophysics and Biochemistry, Yale School of Medicine, Yale University, New Haven, Connecticut, United States of America; Tata Institute of Fundamental Research, INDIA

## Abstract

Microtubules are dynamic cytoskeletal polymers that provide mechanical support for cellular structures, and play important roles in cell division, migration, and intracellular transport. Their intrinsic dynamic instability, primarily controlled by polymerization-dependent GTP hydrolysis, allows for rapid rearrangements of microtubule arrays in response to signaling cues. In neurons, increases in intracellular levels of nicotinamide adenine dinucleotide (NAD^+^) can protect against microtubule loss and axonal degeneration elicited by axonal transection. The protective effects of NAD^+^ on microtubule loss have been shown to be indirect in some systems, for example through the sirtuin-3 pathway. However, it is still possible that NAD^+^ and related metabolites have direct effects on microtubule dynamics to promote assembly or inhibit disassembly. To address this question, we reconstituted microtubule dynamics in an *in vitro* assay with purified bovine brain tubulin and examined the effects of NAD^+^, NADH, and NMN. We found that the compounds had only small effects on the dynamics at the plus and minus ends of the microtubules. Furthermore, these effects were not statistically significant. Consequently, our data support earlier findings that NADs and their precursors influence microtubule growth through indirect mechanisms.

## Introduction

Axonal degeneration, which precedes the death of neurons, marks the onset of many age-related neurodegenerative disorders and axonopathies, including Alzheimer’s disease, multiple sclerosis, and Parkinson’s disease [1–3]. The mammalian nicotinamide mononucleotide adenylyltransferase (NMNAT) isoform 2, and its *Drosophila* ortholog, dNmnat, have been identified as key constituents of an axon survival pathway [4, 5]. NMNATs are nicotinamide adenine dinucleotide (NAD^+^)-synthesizing enzymes that use nicotinamide mononucleotide (NMN) as their substrate. NAD^+^ has several roles in metabolism, acting as a coenzyme in redox reactions, as a donor of ADP-ribose moieties in ADP-ribosylation reactions, and as a precursor of the second messenger molecule cyclic ADP-ribose; consequently, NAD^+^ can regulate cell signaling, gene expression and cell survival [6]. Stimulating NAD^+^ synthesis or decreasing NMN levels has been shown to inhibit axon degeneration and improve mitochondrial function [7–9]. The overexpression of any of the three mammalian isoforms of NMNAT or of WLD^s^, an aberrant fusion protein that contains NMNAT’s enzymatic activity, inhibits degeneration of the distal axonal fragment, following axonal transection [10–13]. Additionally, the overexpression of NMNAT1 protects neurons from degeneration induced by microtubule-destabilizing drugs [14]. In *Drosophila*, dNmnat is also required for axotomy-induced neuroprotection of severed dendritic branches, and its overexpression is sufficient to trigger molecular pathway responsible for this process in the absence of axon injury [15]. Thus, NAD^+^ metabolism is important for neuronal survival.

Depletion of NMNAT2 from axonal compartments after injury is prodegenerative [16]. The subsequent decline in NAD^+^ levels, coupled with the accumulation of NMN, activates SARM1 (sterile alpha and TIR motif containing protein 1) leading to calcium influx, disassembly of the microtubule cytoskeleton, and fragmentation of the detached axon [8, 17, 18]. Because decreased levels of NAD^+^ have been observed in axons undergoing degeneration following both mechanical or chemical insults, it is possible that NAD^+^ and its metabolites could control axonal integrity [9, 14]. Indeed, in explant cultures of dorsal root ganglion (DRG) neurons, exogenously applied NAD^+^ or its precursor, nicotinamide delays axon degeneration after axonal transection [9, 14, 19]. A key question is how NAD^+^ protects neuronal processes from degeneration.

NAD^+^ can protect axons against degeneration and microtubules against depolymerization through activation of NAD^+^-dependent signaling pathways. In cultured DRG neurons, the protective effect of NAD^+^ on axonal degeneration requires sirtuin 1, a nuclear NAD-dependent deacetylase [14]. In cultured breast cancer cells (MCF7) treated with vinblastine, a microtubule-disrupting drug, the protective effect of NAD^+^ on microtubule loss requires sirtuin 3 (SIRT3), a mitochondrial NAD^+^-dependent deacetylase. Additionally, in MCF7 cells NAD^+^ increased microtubule growth, even in cells not treated with vinblastine [19]. Furthermore, activation of SIRT3 by the NAD^+^ precursor nicotinamide riboside protects against noise-induced hearing loss in mice [20]. In contrast to these sirtuin-dependent effects, it has also been shown that NAD^+^ protects against axon degeneration even when administered after axon severing (i.e. in the absence of transcriptional machinery) and in cells from sirtuin-1 knockout mice [9]. This suggests either that other, non-nuclear, signaling pathways are involved or that NAD^+^ might have direct effects on microtubules or other components of the axonal cytoskeleton.

The microtubule cytoskeleton, like the NMNAT pathway, plays key roles in maintaining axonal integrity [4, 5, 12, 13, 21]. For example, fragmentation of the axonal stump following injury requires remodeling and disassembly of the neuronal microtubules [21]. Accordingly, the microtubule-stabilizing drug taxol, similar to NMNAT and NAD^+^, delays axonal degeneration [11, 13, 22–24]. Tubulin, the protein subunit of microtubules, contains a Rossmann fold [25, 26], which forms part of a GTP binding pocket that differs from that of other G-proteins [26]. Interestingly, the βαβ structural motif of the Rossman fold forms part of the dinucleotide-binding site of NAD^+^-dependent enzymes such as the NMNATs [27] and sirtuins [28]. These structural similarities between tubulin and NAD^+^-binding proteins raise the possibility of a direct interaction between tubulin and NAD^+^ (or its reduced form NADH or its precursor NMN). NAD^+^ does not alter the polymerization of purified tubulin in turbidity assays [19]; however, microtubules undergo a process called dynamic instability, characterized by their growth, catastrophe, shrinkage and rescue rates, which leads to changes in microtubule length and number even when total polymer has reached steady state [29]. Therefore, it is possible that NAD^+^ causes more subtle changes in microtubule dynamics. Furthermore, it is possible that NMNAT acts through changes in concentrations of NADH or NMN. Thus, the question whether NAD^+^ and/or its metabolites could directly regulate microtubules in neurons and thereby contribute to delay in axon degeneration remains open.

In this study we use *in vitro* reconstitution assays with purified bovine brain tubulin to test whether the NADs can directly alter microtubule dynamic behavior. We show that NAD^+^ at physiological concentrations has only small effects on microtubule dynamics. Furthermore, similar results were obtained with the reduced version of NAD^+^, NADH, as well as with NMN. Together, our data supports the notion that NAD^+^ and its precursors regulates microtubule dynamics through indirect mechanisms.

## Results and discussion

**Fig 1:**
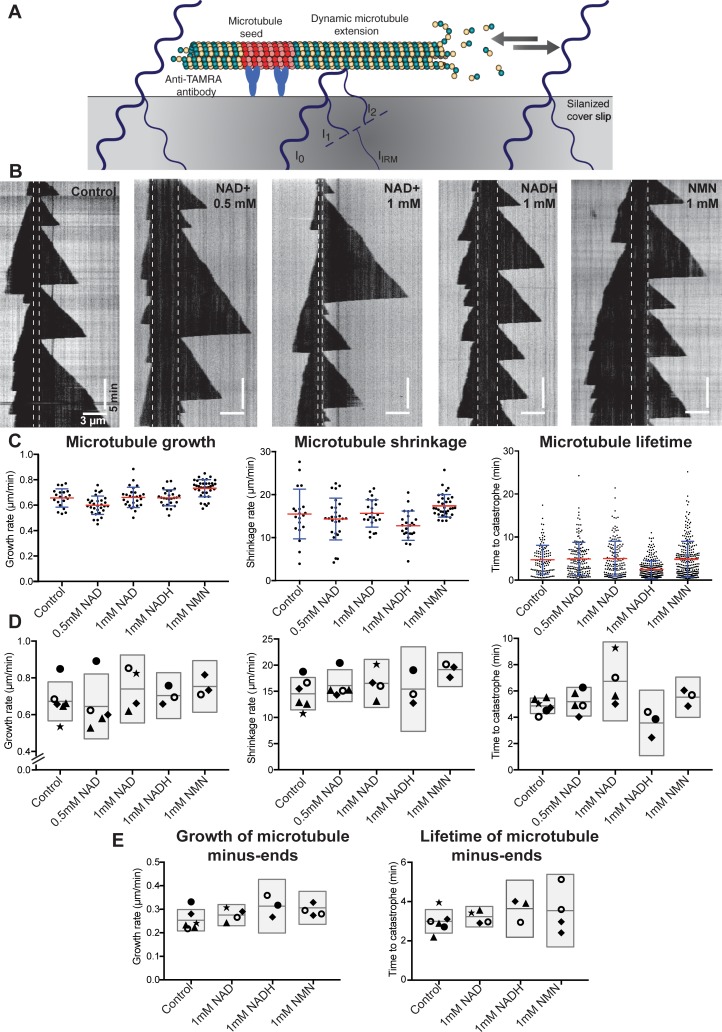
The effect of NADs on microtubule dynamics *in vitro*. **(A)** Schematic of an IRM *in vitro* microtubule assay. Dynamic, label-free microtubules were grown from TAMRA-labeled GMPCPP-stabilized microtubule seeds attached to a silanized coverslip through anti-TAMRA antibodies. Microtubule dynamics was observed by time-lapse IR microscopy. Microtubule plus ends (marked with gray arrows) and minus ends were analyzed in this assay. Illumination light (blue line) is reflected from the glass/water interface and water/microtubule interface. Microtubule image is formed by the interference of reflected light. I_IRM_: interference intensity; I_0_: incident light intensity; I_1_: intensity of light reflected of glass/sample interface; I_2_: intensity of light reflected from water/microtubule interface (B) Representative kymographs depicting dynamic behavior of individual microtubule polymers in the presence or absence of NADs or NMN (as specified in the top right corner). Dashed lines indicate the position of TAMRA-labeled microtubule seeds. For all kymographs, microtubule plus-end is positioned to the right of the seed and corresponds to the orientation of microtubule on the illustration in panel (A) Horizontal scale bars, 3μm. Vertical scale bars, 5 minutes. (C) Scatter plots representing the effect of NADs and NMN on parameters of microtubule dynamic instability for an individual experimental repeat. Points on the diagrams depicting microtubule growth and shrinkage rates correspond to the average growth or shrinkage rate of individual microtubule within the sample. Time to catastrophe represents the lifetime of all analyzed events within the repeat. Red line indicates the average, blue error bars correspond to standard deviation (SD). The exact values for parameters of microtubule dynamic instability plotted here can be found in S1 Table in bold. (D) Plots representing averages for all individual experiments. Same shape symbols correspond to the experiments performed side-by-side on the same day. Detail values for depicted averages are combined in S1 Table. (E) Plots representing average growth rates and time to catastrophe measured for microtubule minus-ends. Averages for all individual experiments are combined in S2 Table. Shaded boxes in panels D and E represents 95% confidence intervals with middle lines corresponding to the averages calculated based on the experimental repeats.

To study the effect of NAD^+^ and its metabolites on microtubule dynamics *in vitro*, we grew unlabeled GTP-tubulin extensions from TAMRA-labeled, GMPCPP-stabilized microtubule seeds attached to a functionalized coverslip (Fig 1A) [30]. The growth and shrinkage of microtubule polymers was imaged using interference reflection microscopy (IRM) [31]. Microtubule dynamics was illustrated using kymographs: stacks of intensity profiles measured along a microtubule in the consecutive frames of a time-lapse microscopy image (Fig 1B). Kymographs facilitated measurement of the average growth and shrinkage rates for individual microtubules, as well as the times to catastrophe (Fig 1B, S1 Table), the time a microtubule spends growing before it transitions to shrinkage. The time to catastrophe, or microtubule lifetime is the inverse of microtubule catastrophe frequency. From here on, we use rates of microtubule growth and shrinkage, and time to catastrophe, when referring to measurements of plus end dynamics, unless specified otherwise.

The average microtubule growth rate for 7.5 μM tubulin was 0.67 ± 0.04 μm/min (mean ± SEM, *n* = 6 experiments with 869 growth events); the average microtubule shrinkage rate was 14.54 ± 1.21 (mean ± SEM, *n* = 6 experiments with 448 shrinkage events); and average time to catastrophe was 4.87 ± 0.23 minutes (mean ± SEM, *n* = 6 experiments with 1020 catastrophes) (Fig 1D, S1 Table).

We then asked whether NAD^+^, within the physiological range of concentrations, can directly regulate microtubule dynamics. The intracellular levels of NAD^+^ vary depending on the type of the organism and its age [7, 32]. Moreover, the NAD^+^ concentration can change up to 2-fold in response to glucose depravation or caloric restriction. However, it is usually maintained at 0.2–0.5 mM [32–35]. Extracellular addition of 5 mM NAD^+^, sufficient to promote physiological effects, increases the intracellular concentration two-fold [19, 34]. Thus, to verify the ability of NAD^+^ to regulate microtubule dynamics, we added 0.5 mM or 1 mM NAD^+^ to our *in vitro* assay and monitored the behavior of the microtubules. The effects of NAD^+^ on microtubule growth and shrinkage rates were small and not statistically significant. The growth rates for 0.5 mM and 1 mM NAD^+^ were 0.65 ± 0.06 μm/min (SEM, *n* = 5 experiments with 720 growth events) and 0.74 ± 0.06 μm/min (SEM, *n* = 4 experiments with 453 growth events), respectively, corresponding to 3% and 10% increases (Fig 1C and 1D, S1 Table). The shrinkage rates were 16.1 ± 1.1 μm/min (SEM, *n* = 5 with 323 shrinkage events) and 16.5 ± 1.4 μm/min (SEM, *n* = 4 with 259 shrinkage events), corresponding to 10% and 13% increases (Fig 1C and 1D, S1 Table). When the NAD+ results were compared with matched controls, these differences were not statistically significant by a non-parametric test (see S1 Table).

The effect of NAD^+^ on microtubule lifetimes was larger than on growth and shrinkage, though still not statistically significant. The mean microtubule lifetimes for 0.5 mM and 1 mM NAD^+^ were 5.19 ± 0.39 min (SEM, *n* = 5 experiments with 821 catastrophes) and 6.74 ± 0.94 min (SEM, *n* = 4 experiments with 507 catastrophes), respectively, corresponding to 6.5% and 38% increases (Fig 1C and 1D, S1 Table). When the NAD^+^ results were compared with matched controls, these differences were not statistically significant by a non-parametric test (see S1 Table). Though the results are not statistically significant, there could nevertheless be an effect of NAD^+^ on lifetime: if we pool the data at 1 mM NAD^+^ and compare to the pooled data for the controls, then the increase is 31% ± 3%.

While most of the NADs exists in the cell in an oxidized NAD^+^ form, in redox reactions NAD^+^ serves as an acceptor of electrons resulting in its reduction to NADH [6]. The intracellular concentration of NADH is lower than NAD^+^, and typically maintained at 50–160 μM [32, 36] however, it can reach up to 0.89 mM in some cytoplasmic compartments [37]. Therefore, we conducted the dynamic assay in the presence of 1 mM NADH to test whether the redox potential of NADs can affect their ability to regulate microtubule dynamics. We checked that our NAD^+^ and NADH solutions were in the expected oxidation state by measuring their absorbance spectra (NADH has a prominent peak at 339 nm). We found that NADH had only small effects on microtubule growth rate, shrinkage rate and lifetime. The values were 0.70 ± 0.03 μm/min (SEM, *n* = 3 experiments with 488 events), 15.4 ± 1.9 μm/min (SEM, *n* = 3 experiments with 211 events), and 3.6 ± 0.6 min (SEM, *n* = 3 experiments with 708 events) respectively (Fig 1C and 1D, S1 Table). These correspond to a 5% increase in growth rate, a 6% increase in shrinkage rate, and 26% decrease in lifetime. When the NADH results were compared with matched controls, these differences were not statistically significant by a non-parametric test (see S1 Table). Though the effect on lifetime is not statistically significant, NADH could nevertheless affect lifetime: if we pool the data at 1 mM NADH and compare to the pooled data for the controls, then the decrease is 27% ± 5%.

The cellular NAD^+^ pool comes from the turnover of NAD^+^/NADH in a salvage pathway that recycles components of the NAD^+^/NADH molecules, or de novo biosynthesis [32]. Both of these pathways converge at the reactions catalyzed by NMNATs that result in formation of NAD^+^. NMN is a key substrate for the reactions in the dominant salvage pathway and it is universal for all cellular compartments [6, 32]. Remarkably, recent studies implicate elevated NMN concentration in cellular dysfunction and axon degeneration observed during aging and in neurodevelopmental disorders [8, 16, 18]. Therefore, we analyzed the effect of the NAD^+^ precursor, NMN at 1 mM concentration on dynamic microtubules. We found that NMN had only small effects on microtubule growth rate, shrinkage rate and lifetime. The values were 0.75 ± 0.03 μm/min (SEM, *n* = 3 experiments with 619 events), 19.0 ± 0.72 μm/min (SEM, *n* = 3 experiments with 320 events), and 5.53 ± 0.36 min (SEM, *n* = 3 experiments with 713 events) respectively (Fig 1C and 1D, S1 Table). These correspond to a 12% increase in growth rate, a 31% increase in shrinkage rate, and 13% increase in lifetime. When the NMN results were compared with matched controls, these differences were not statistically significant by a non-parametric test (S1 Table). Though the effect of NMN on shrinkage is not statistically significant, there could nevertheless be an effect: if we pool the data at 1 mM NMN and compare to the pooled data for the controls, then the increase is 30% ± 3%.

Although in many cell types microtubules are nucleated at the microtubule organizing center and extend with their plus ends towards the cell periphery, in neurons this centralized organization is lost during maturation. Therefore, minus ends of microtubules in differentiated neurons are not anchored in the centrosome, and are frequently found free in the cytoplasm [21]. The presence of acentrosomal microtubules in neurons, with their minus ends accessible to regulators, prompted us to investigate whether NADs could alter minus end dynamics. The average growth rate of microtubule minus end measured in our *in vitro* assay was 0.25 ± 0.02 μm/min (SEM, *n* = 6 experiments with 94 growth events). The addition of 1 mM NAD^+^, NADH, or NMN had no statistically significant effect on the growth rate of microtubule minus end in the assay conditions, and resulted in the following rates 0.28 ± 0.01 μm/min (SEM, *n* = 4 experiments with 67 growth events), 0.31 ± 0.03 μm/min (SEM, *n* = 3 experiments with 34 growth events), and 0.29± 0.01 μm/min (SEM, *n* = 4 experiments with 62 growth events), respectively (Fig 1E, S2 Table). Similar to minus end growth rate, we observed no statistically significant effect of NADs or NMN on microtubule minus end lifetime (Fig 1E, S2 Table). In addition to dynamics at microtubule plus and minus ends, we did not observe microtubule severing. Thus, our data indicates that NADs and NMN does not alter minus end microtubule dynamics.

Our experiments show that NAD^+^, NADH and NMN have only small direct effects on plus and minus end microtubule dynamics in *in vitro* reconstitution assays. When compared with matched controls, none of the effects were statistically significant. The magnitudes of most of the effects were small, namely 10% or less. The only larger effects were for NAD^+^ on lifetime (31% increase), NADH on lifetime (27% decrease) and NMN on shrinkage rate (30% increase). Thus, we conclude that none of these metabolites have pronounced effects on microtubule dynamics i*n vitro*: none of the effects are statistically significant and it is unlikely that any give rise to changes larger than 50%. The relatively small effects of NAD^+^ on microtubule dynamics is consistent with Harkcom *et al*. (2014) who found no effect of 10 mM NAD^+^ on microtubule growth in a turbidity assay. Together our data indicates that NAD^+^ and its metabolites are unlikely to be direct regulators of microtubule dynamic instability *in vitro*. Instead, our results favor the hypothesis that changes in microtubule dynamics observed prior to axon degeneration result from altered enzymatic activity of microtubule regulators in response to changes in intracellular levels of NAD^+^ and/or NMN, as shown in earlier work [14, 19, 20].

## Material and methods

### Dynamic microtubules assay

Tubulin was purified using two cycles of polymerization and depolymerization in high molarity buffer from bovine brains as previously described [38]. The dynamic microtubule assays were performed in microchannels prepared from two silanized coverslips sealed with two strips of parafilm (Bemic NA, Neenah, WI) [30, 39]. GMPCPP-stabilized microtubule seeds labeled with TAMRA dye (ThermoFisher Scientific, Waltham, MA) were used to initiate the growth of dynamic microtubule extensions from unlabeled GTP-tubulin. The dynamic microtubules were visualized using interference reflection microscopy [31] in the assay buffer (80mM K-PIPES, 1mM EGTA, 1mM MgCl_2_, pH 6.9, 1mM DTT, 2mM GTP). NAD^+^, NADH (Roche, Indianapolis, IN) or NMN (Sigma-Aldrich Corp, St. Louis, MO) was mixed with the reaction buffer prior to flowing into the imaging chamber. The time-lapse images were collected with a sCMOS camera (Zyla 4.2, Andor, Belfast, Scotland) mounted on a Ti Eclipse Nikon microscope using a 100x/1.49 NA Apochromat objective (Nikon, Melville, NY). All dynamic assays were imaged at 0.2 fps rate for 40 minutes at 34°C.

### Image analysis

Image analysis was performed using Fiji analysis software [40]. Briefly, the background images, generated by averaging 32 images of empty reaction channels were subtracted from the corresponding time series to enhance contrast [31]. Subsequently, kymographs of individual microtubules were generated and microtubules growth and shrinkage velocities, as well as catastrophe frequencies were measured. To calculate growth rates, only events that lasted at least 75 seconds were taken into consideration, whereas for shrinkage rates events that were observed for at least 2 consecutive frames (10 seconds) were analyzed. Next, the average growth and shrinkage rates per microtubule were calculated. We followed the same rules to measure growth rates and lifetime of microtubule minus-ends, except to calculate growth rates of minus-ends, events that lasted at least 60 seconds were included in analysis. However, we were unable to determine shrinkage rates of microtubule minus-ends due to limited temporal resolution. Statistical analysis for all individual experiments and repeats was performed using GraphPad Prism7 or R statistical software [41]. The averages of all tested conditions were compared with their respective controls using Wilcoxon signed-rank test and the variability between the same treatments on subsequent days was determined using unpaired student’s t-test with Bonferroni correction.

## Supporting information

S1 Table Parameters of microtubule plus-end dynamics for all experimental repeats. For each microtubule there were several individual events measured (see Fig 1B). The means and SDs of the growth and shrinkage rates were calculated for *n* equal to the number of microtubules. For the time to catastrophe, *n* equals the number of events. Values in bold correspond to the graph in Fig 1C. *p* -values are for the Wilcoxon signed-rank test on the means (compared to the respective controls). ns (not significant), SD (standard deviation), MT (microtubule).
(PDF)

S2 Table Growth rates and lifetimes of microtubule minus-ends. For each experimental repeat, 10 microtubules were tracked for 10 minutes to determine lifetime and growth rate of microtubule minus-ends. For each microtubule several individual events were measured. The means and SDs of the growth rates were calculated for *n* equal to the number of microtubules. For the time to catastrophe, *n* equals the number of events. *p*-values are for the Wilcoxon signed-rank test on the means (compared to the respective controls). ns (not significant), SD (standard deviation), MT (microtubule). *Number of microtubules reported in the table corresponds to the microtubules that displayed dynamic behavior of the minus-end within the conditions of analysis and their growth lasted at least 60 seconds (see methods sections for more details).
(PDF)
