# Supplementary material for: Nicotinamide adenine dinucleotides and their precursor NMN have no direct effect on microtubule dynamics in purified brain tubulin
Source: PLoS One. 2019 Aug 8;14(8):e0220794. doi: 10.1371/journal.pone.0220794 (PMC6687165; doi:10.1371/journal.pone.0220794)
Supplement: S1 Table — For each microtubule there were several individual events measured (see Fig 1B). The means and SDs of the growth and shrinkage rates were calculated for n equal to the number of microtubules. For the time to catastrophe, n equals the number of events. Values in bold correspond to the graph in Fig 1C. p -values are for the Wilcoxon signed-rank test on the means (compared to the respective controls). ns (not significant), SD (standard deviation), MT (microtubule). (PDF) [file pone.0220794.s001.pdf]

Sample	Repeat	Growth rate (µm/min)			Shrinkage rate (µm/min)			Time to catastrophe (min)		
		Growth rate (µm/min) ± SD	Number of MTs (events) analyzed	Wilcoxon signed-rank test *p* value	Shrinkage rate (µm/min) ± SD	Number of MTs (events) analyzed	Wilcoxon signed-rank test *p* value	Time to catastrophe (min) ± SD	Number of MTs (events) analyzed	Wilcoxon signed-rank test *p* value
Control (untreated)	1	0.5348 ± 0.063	20 (127)		10.78 ± 2.945	20 (69)		5.04 ± 4.098	20 (120)	
	2	0.6617 ± 0.074	26 (127)		12.61 ± 4.663	26 (78)		5.523 ± 4.413	26 (140)	
	3	0.6469 ± 0.051	33 (180)		12.93± 2.597	33 (112)		5.389 ± 4.627	33 (212)	
	4	0.6841 ± 0.072	33 (184)		16.65 ± 3.298	33 (79)		4.037 ± 3.295	33 (226)	
	**5**	**0.6456 ± 0.072**	**20 (107)**		**15.49 ± 5.788**	**20 (49)**		**4.729 ± 3.348**	**20 (125)**	
	6	0.8485 ± 0.12	29 (144)		18.75 ± 6.384	29 (61)		4.515 ± 4.621	29 (197)	
0.5 mM NAD	1	0.5285± 0.058	36 (152)		15.31 ± 3.97	36 (71)		4.888 ± 3.481	36 (165)	
	2	0.5803 ± 0.059	27 (175)		15.27 ± 2.73	27 (75)		4.029 ± 3.695	27 (236)	
	3	0.6237 ± 0.118	24 (106)	0.125 (ns)	15.11 ± 5.771	24 (38)	0.3125 (ns)	5.841 ± 5.333	24 (106)	0.625 (ns)
	**4**	**0.5995 ± 0.073**	**30 (149)**		**14.32± 4.861**	**30 (66)**		**4.919 ± 3.88**	**30 (176)**	
	5	0.8911 ± 0.113	31 (138)		20.43 ± 8.05	31 (73)		6.266 4.475	31 (138)	
1 mM NAD	1	0.825 ± 0.105	20 (78)		20.18 ± 6.149	20 (46)		9.277 ± 7.151	20 (80)	
	2	0.6201 ± 0.064	29 (143)	0.375 (ns)	13.15 ± 3.016	29 (77)	0.125 (ns)	5.647 ± 4.45	29 (158)	0.125 (ns)
	3	0.853 ± 0.071	21 (96)		16.03 ± 5.071	21 (64)		7.01 ± 5.346	21 (104)	
	**4**	**0.6611 ± 0.08**	**27 (136)**		**16.64 ± 5.871**	**27 (72)**		**5.01 ± 4.041**	**27 (165)**	
1mM NMN	1	0.7094 ± 0.085	53 (260)		20.16 ± 4.265	53 (135)		5.686 ± 4.684	53 (288)	
	**2**	**0.7332 ± 0.068**	**37 (144)**	0.25 (ns)	**17.67 ± 3.083**	**37 (121)**	0.250 (ns)	**4.846 ± 4.157**	**37 (267)**	0.250 (ns)
	3	0.8168 ± 0.079	30 (215)		19.13 ± 5.824	30 (64)		6.049 ± 4.398	30 (158)	
1 mM NADH	1	0.695 ± 0.074	38 (181)		14.46 ± 3.911	38 (93)		4.4 ± 3.842	38 (241)	
	**2**	**0.658 ± 0.063**	**27 (170)**	>0.9999 (ns)	**12.77 ± 3.404**	**27 (63)**	0.500 (ns)	**2.456 ± 2.082**	**27 (272)**	0.500 (ns)
	3	0.7574 ± 0.096	27 (137)		19.05 ± 3.701	27 (55)		3.861 ± 4.312	27 (195)	
