# Supplementary material for: Nicotinamide adenine dinucleotides and their precursor NMN have no direct effect on microtubule dynamics in purified brain tubulin
Source: PLoS One. 2019 Aug 8;14(8):e0220794. doi: 10.1371/journal.pone.0220794 (PMC6687165; doi:10.1371/journal.pone.0220794)
Supplement: S2 Table — For each experimental repeat, 10 microtubules were tracked for 10 minutes to determine lifetime and growth rate of microtubule minus-ends. For each microtubule several individual events were measured. The means and SDs of the growth rates were calculated for n equal to the number of microtubules. For the time to catastrophe, n equals the number of events. p-values are for the Wilcoxon signed-rank test on the means (compared to the respective controls). ns (not significant), SD (standard deviation), MT (microtubule). *Number of microtubules reported in the table corresponds to the microtubules that displayed dynamic behavior of the minus-end within the conditions of analysis and their growth lasted at least 60 seconds (see methods sections for more details). (PDF) [file pone.0220794.s002.pdf]

Sample	Repeat	Growth rate (µm/min)			Time to catastrophe (min)		
		Growth rate (µm/min) ± SD	Number of MTs* (events) analyzed	Wilcoxon signed-rank test *p* value	Time to catastrophe (min) ± SD	Number of events analyzed	Wilcoxon signed-rank test *p* value
Control (untreated)	1	0.2412 ± 0.048	10 (20)		3.958 ± 1.936	16	
	2	0.2233 ± 0.059	6 (14)		2.896 ± 1.085	12	
	3	0.2333 ± 0.021	7 (13)		2.197 ± 1.549	11	
	4	0.2174 ± 0.062	6 (14)		2.992 ± 1.526	11	
	5	0.2802 ± 0.057	8 (20)		3.106 ± 1.402	15	
	6	0.3314 ± 0.071	29 (13)		2.713 ± 1.959	9	
1 mM NAD	1	0.3073 ± 0.092	10 (19)	0.125 (ns)	3.429 ± 2.268	13	0.875 (ns)
	2	0.2425 ± 0.057	8 (17)		3.567 ± 1.69	15	
	3	0.2655 ± 0.072	7 (15)		2.968 ± 1.446	13	
	4	0.2902 ± 0.089	10 (16)		2.905 ± 2.583	14	
1mM NMN	1	0.2803 ± 0.095	4 (7)	0.25 (ns)	3.597 ± 3.847	6	0.875 (ns)
	2	0.2952 ± 0.1023	5 (10)		5.125 ± 2.568	6	
	3	0.3288 ± 0.093	10 (24)		2.413 ± 1.615	21	
	4	0.2739 ± 0.055	9 (21)		2.974 ± 1.956	16	
1 mM NADH	1	0.3588 ± 0.163	7 (12)	> 0.999 (ns)	2.947 ± 1.828	11	0.500 (ns)
	2	0.2668 ± 0.054	6 (10)		4.012 ± 2.827	7	
	3	0.3174 ± 0.05	5 (12)		3.917 ± 1.664	10	
